# Assessment of the Feasibility of a Future Integrated Larger-Scale Epidemiological Study to Evaluate Health Risks of Air Pollution Episodes in Children

**DOI:** 10.3390/ijerph19148531

**Published:** 2022-07-12

**Authors:** Sarah J. D. Nauwelaerts, Koen De Cremer, Natalia Bustos Sierra, Mathieu Gand, Dirk Van Geel, Maud Delvoye, Els Vandermassen, Jordy Vercauteren, Christophe Stroobants, Alfred Bernard, Nelly D. Saenen, Tim S. Nawrot, Nancy H. C. Roosens, Sigrid C. J. De Keersmaecker

**Affiliations:** 1 Transversal Activities in Applied Genomics, Sciensano, 1050 Brussels, Belgium; sarah.nauwelaerts@sciensano.be (S.J.D.N.); mathieu.gand@sciensano.be (M.G.); dirk.vangeel@sciensano.be (D.V.G.); maud.delvoye@sciensano.be (M.D.); els.vandermassen@sciensano.be (E.V.); nancy.roosens@sciensano.be (N.H.C.R.); 2 Centre for Toxicology and Applied Pharmacology, University Catholique de Louvain, 1200 Brussels, Belgium; alfred.bernard@uclouvain.be; 3 Platform Chromatography and Mass Spectrometry, Sciensano, 1050 Brussels, Belgium; koen.decremer@sciensano.be; 4 Epidemiology and Public Health, Sciensano, 1050 Brussels, Belgium; natalia.bustossierra@sciensano.be; 5 Unit Air, Vlaamse Milieumaatschappij, 2000 Antwerpen, Belgium; j.vercauteren@vmm.be (J.V.); c.stroobants@vmm.be (C.S.); 6 Centre for Environmental Sciences, Hasselt University, 3590 Diepenbeek, Belgium; nelly.saenen@uhasselt.be (N.D.S.); tim.nawrot@uhasselt.be (T.S.N.); 7 Department of Public Health and Primary Care, KU Leuven, 3000 Leuven, Belgium

**Keywords:** biomarkers, epidemiological studies, non-invasive, children, feasibility, air pollution, particulate matter, ozone

## Abstract

Air pollution exposure can lead to exacerbation of respiratory disorders in children. Using sensitive biomarkers helps to assess the impact of air pollution on children’s respiratory health and combining protein, genetic and epigenetic biomarkers gives insights on their interrelatedness. Most studies do not contain such an integrated approach and investigate these biomarkers individually in blood, although its collection in children is challenging. Our study aimed at assessing the feasibility of conducting future integrated larger-scale studies evaluating respiratory health risks of air pollution episodes in children, based on a qualitative analysis of the technical and logistic aspects of a small-scale field study involving 42 children. This included the preparation, collection and storage of non-invasive samples (urine, saliva), the measurement of general and respiratory health parameters and the measurement of specific biomarkers (genetic, protein, epigenetic) of respiratory health and air pollution exposure. Bottlenecks were identified and modifications were proposed to expand this integrated study to a higher number of children, time points and locations. This would allow for non-invasive assessment of the impact of air pollution exposure on the respiratory health of children in future larger-scale studies, which is critical for the development of policies or measures at the population level.

## 1. Introduction

Air pollution is one of the biggest environmental hazards for public health and a major risk factor for acute and chronic effects on the respiratory tract [1,2]. Reduced air quality is, for the most part, caused by anthropogenic activities. It includes increased particulate matter (PM) and ozone levels, which are some of the most problematic air pollutants [3]. Several studies have shown their adverse effects occurring at exposure levels commonly encountered around those mentioned by the WHO guidelines applicable at the time of the study [4]. The thresholds in these guidelines are calculated for the entire population. However, special consideration should be given to determine appropriate levels applicable for vulnerable groups, especially children [5]. Indeed, air pollution represents a major threat for them. It can be an increasing factor for respiratory health problems, such as increased lung function impairment [6,7,8], respiratory diseases [9,10,11,12,13], enhancement of allergic reactions and airway inflammation [14,15]. It also leads to increased blood pressure with associated risk of future hypertension [16]. To investigate how and at what level air pollution affects the respiratory health of children, population-based field studies are important to conduct. Only a limited number of large-scale studies have been conducted in children, as the design and execution demands of such experiments are relatively high in regards to manpower, time and cost. Nevertheless, more results of studies in children should become available.

When conducting these types of studies in adults, the investigation of biomarkers, defined as indicators of exposure, effect and susceptibility, is usually done in blood. However, this biofluid remains difficult to collect in children as it may represent an issue for the parents and the research ethics committee. Therefore, there is a need for a study setup for children that uses non-invasive samples. This study design should involve the appropriate collection, storage and downstream processing of suitable non-invasive samples, such as urine and saliva, by using adequate technologies to analyze biomarkers in large-scale studies. Different types of biomarkers exist at protein, genetic and epigenetic levels. When their investigation is combined, they give additional insights and complementary information on their interrelated and complementary relationships.

The club cell secretory protein (hereafter referred to as CC16) is an interesting protein biomarker as it has been described as an early marker of pulmonary injury and permeability and respiratory diseases [17,18]. Recently, as an alternative to serum CC16, urinary CC16 (U-CC16) was measured using a validated multiple reaction monitoring (MRM) method. This allowed the high-throughput, and hence cost-effective, relative quantification of U-CC16, urinary beta-2-microglobulin (U-β2M) and urinary retinol binding protein 4 (U-RBP4) [19]. The latter are two potential adjusters for the variations in diuresis and renal handling of proteins. CC16 has been investigated in urine, as a potential proxy of serum CC16, where changing concentrations were observed following environmental exposures [20,21,22], such as PM exposure [23], and where altered levels of U-CC16 have also been associated with respiratory disorders [24,25].

Some specific single nucleotide polymorphisms (SNPs) have also been described as potential risk factors of respiratory health. These can be used as genetic biomarkers. Two such interesting ones are the SNP *CC16* G38A and the SNP uteroglobin-related protein 1 (*UGRP*1) G112A. Both were found to be associated with the development of hypersensitive response or respiratory health conditions [26,27,28,29,30]. Moreover, the genotype can sometimes influence the associated phenotype. Carriers of the A-allele of the SNP *CC16* G38A were associated with lower circulating CC16 levels and with increased odds of asthma [31]. These results show the importance of combining the measurement of protein and genetic biomarkers. For investigation of this type of genetic biomarker in larger-scale epidemiological studies, saliva or buccal cells have been shown to be the preferred non-invasive sample sources. These samples yield high quantities of high-quality DNA, allowing a cost-effective setup to investigate multiple SNPs in numerous samples. Recently, DNA obtained from buccal cells was used to investigate the SNP *CC16* G38A using a qPCR-based method to demonstrate that the A-allele was associated with lower urinary CC16 levels [23,32,33].

Lately, the epigenome was also found to be an important target of air pollution, where epigenetic variations contributed to increased disease susceptibility [34,35,36]. A few prospective studies, using saliva as source, have found genes, such as the ten-eleven translocation methyl cytosine dioxygenase 1 (*TET-1*) gene, to be differentially methylated following air pollution exposure [37]. With pyrosequencing, a real-time sequencing method and considered a “gold standard”, quantitative allele quantification at single base resolution can be obtained. In addition, specific micro RNAs (miRNAs) are influenced by PM exposure and can be measured, especially when protected in the extracellular environment through microvesicles or exosomes [38]. Amongst them are miR-146a and miR-222, both shown to be responsive to PM exposure in adults [39,40,41,42]. They could be interesting biomarkers to measure in exosome-containing fluids, such as saliva, during the monitoring of air pollution exposure. They can easily be measured with RT-qPCR-based methods [42].

Recently, the use of integrated studies with a multidimensional approach was shown to lead to a better knowledge of certain disorders or exposure impacts [43,44,45]. Only a limited number of studies have investigated the combination of different types of biomarkers in the context of respiratory health and air pollution. Furthermore, very few studies investigated the technical requirements and bottlenecks associated with this type of large-scale study involving non-invasive samples and the analysis of numerous samples. Requirements at the level of sample conservation, quality and quantity as well as the available technologies to measure the different biomarkers in a cost-effective setup should be evaluated.

The aim of this study was to evaluate, at a qualitative level, the technical and logistic feasibility of conducting this type of integrated approach. To this end, we designed and conducted a small-scale pilot field study in children exposed to different levels of air pollution. We considered the examination of the children in the most appropriate setup and location, including the methods to monitor their air pollution exposure. Additionally, we investigated the type of health parameters and different biomarkers to measure in the most adequately selected, collected and appropriately stored non-invasive samples with cost-effective technologies. These investigated biomarkers are sensitive indicators of airway damage and inflammation (protein biomarkers) and of genetic and epigenetic variations. In addition, a general health parameter (blood pressure) and parameters of respiratory health, i.e., fractional exhaled nitric oxide (FeNO) and lung function (spirometry), were measured through onsite examination. A questionnaire was used to collect information on the health status of the child. The most efficient integrated approach was proposed. Additionally, as part of the optimal design of future larger-scale epidemiological studies in children, we also suggested the necessary adaptations to make from the initially proposed workflow to give a maximal result with minimal effort.

## 2. Materials and Methods

**Figure 1:**
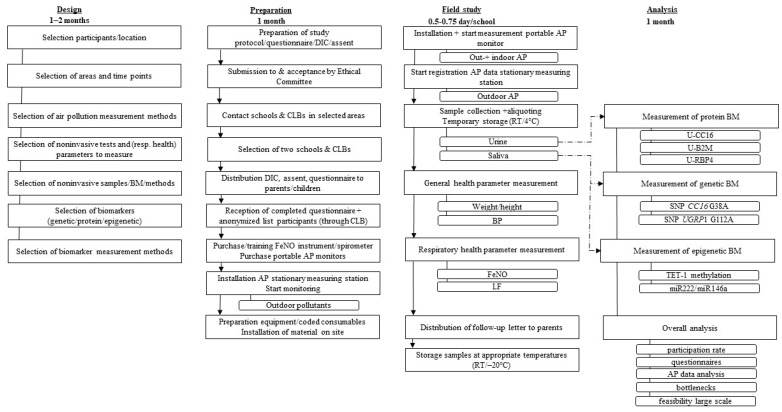
Flowchart of the feasibility study, comprising the several steps of design, preparation, the orchestration of the field study and the analysis of the measured parameters, including the time frame required for each of these steps. AP: air pollution; β2M: beta-2-microglobulin; BM: biomarker; BP: blood pressure; CC16: club cell protein; CLB: pupil guidance center, DIC: document of informed consent; FeNO: fractional exhaled nitric oxide; LF: lung function; RT: room temperature; SNP: single nucleotide polymorphism; TET-1: ten-eleven translocation methylcytosine dioxygenase 1; UGRP1: uteroglobin-related protein.

A flow diagram of the field study is shown in Figure 1. It illustrates the different steps followed for the design, the preparation, and the orchestration of the field study as well as the subsequent analysis of the measured parameters. Before the field study, a test phase was conducted to identify a first set of bottlenecks in the workflow of the study, without performing any air pollution measurements. A number of these hurdles were taken into account before starting with the actual field study. A detailed overview of the whole procedure used in the field study and the test phase is schematized in Supplementary Figure S1.

### 2.1. Field Study Design

This pilot study aimed to examine a limited number of subjects to allow for the estimation of critical practical bottlenecks and strengths of the selected methods and design. The design of the study contained several phases. Firstly, the location to target the vulnerable population, i.e., children aged 9 to 11 years old, was defined. Secondly, the types of non-invasive samples as well as the appropriate cost-effective methods were selected to measure the different relevant biomarkers at different levels. Thirdly, practical aspects were investigated for the measurement of contrasting levels of the most problematic pollutants (PM and ozone). Fourthly, two types of study designs were envisaged: a one-point study (such as cross-sectional studies) and a two-point study (such as longitudinal studies). Taking into account that the focus of this study was more on feasibility related to the logistic aspects of conducting such a study in children and less on the obtained outcomes at the level of the parameters and biomarkers, no power or sample size calculations were done. Nevertheless, we did conduct small power simulations to compare a one-point versus a two-point study design. Assuming population parameters based on plausible values as obtained from this field study, we could conclude, as expected, that the two-point study design was found to yield a higher simulated power. It reduces intersubject variability by using paired data obtained from measuring each subject twice (results not shown). The two-point study design was therefore selected.

### 2.2. Field Study Preparation

Once the design was set, a detailed study protocol was established. It describes the followed methodology and procedures, the examinations and the laboratory investigations, and included the established documents, such as a questionnaire, a follow-up letter, a document of informed consent (DIC) and an assent. It also included information on safety management issues and data management. This multi-phase study was submitted to and approved by the Ethics committee of the Cliniques universitaires Saint-Luc (Registration number B403201734310).

In the context of the recruitment of children, it was decided to contact ten primary schools by email and with a follow-up telephone call. Head-teachers of three schools consented to participate. Two schools, located in areas with contrasting air pollution expectancies, were finally selected. Two to three weeks before the start of the field study, information letters, documents of informed consent (DIC) and assents, i.e., a simplified adapted graphical version of the DIC (see Supplementary Figure S2), were disseminated to all parents through the class teachers of the selected schools. Parents were asked to fill in a questionnaire addressing the social and medical background of the children and their family as well as their in- and out-of-house environment. The questions were partially retrieved from a questionnaire previously used in the context of a similar study, involving young children and their respiratory health [46,47]. Amongst them were questions inquiring about general and respiratory health issues that might have occurred in the last twelve months before the study. At the end of the study, a follow-up letter was distributed to the parents of all children, including those who did not participate. This allowed them to give their feedback on the study, on their reasons for non-participation and on their willingness to potentially participate again in this type of study. The completed documents were collected by the teachers and transferred to the CLB, a ‘pupil guidance center’ specialized in, amongst others, preventive health care and which is linked with specific Dutch-speaking schools in Belgium. The CLB anonymized the content before transferring them back to the research team. In the week before the start of the field study, all sample collection material was labelled using the same coded system as on the consent forms and questionnaires, ensuring the anonymity of the children. Instruments needed for measuring FeNO, lung function and blood pressure were acquired and training was organized for the use of them. The setup for the air pollution measurement was finalized (see more details in the Material & Methods: Air pollution measurement section).

The test phase was conducted in a primary school on 9th of March 2018, located in the Flemish Brabant region in Belgium. The field study took place at two time points occurring in the summer and winter, i.e., on the 11th of September 2018 and the 7th of January 2019, in a primary school in the urban area of Brussels and, with a three-day interval, on the 14th of September 2018 and the 10th of January 2019 in a primary school in a rural area in Limburg. A total of 36 children from the urban school and 56 children form the rural school were invited to participate in the study.

### 2.3. Examinations and Sample Collection

The field work was performed by members of the Sciensano research team and medical staff members of the CLB, including one doctor. The non-invasive samples that were collected and the parameters that were measured are summarized in Figure 1 and in more detail in Supplementary Figure S1.

Because of the relatively limited staff available, the field study took place on two different days in the urban and rural school, with a maximum of three days between both. The staff present during the field study on one day at one school comprised of 8 people. Two medical staff members of the CLB identified the participating children, labelled them with a unique identification code to ensure anonymity towards the research team and measured the general health parameters. Six members of the research team were needed for the sampling and the measurement of FeNO and lung function. On the same day, an additional research team member was present in the other school to collect additional urine samples in order to compare the outcomes between both schools as their studies were conducted on different days. Small groups of children were called out of the classes and guided to a room specifically dedicated for the examinations required for this field study.

#### 2.3.1. Urine Collection

A “second morning” urine sample of each child was collected between 9 am and 12 am with a 125 mL sample vessel (VWR, Leuven, Belgium). The samples were immediately aliquoted on site and maintained in a cooled container until final storage later that day. Urine samples should be stored at −20 °C or at −80 °C for prolonged storage, which was the case here. Additionally, as the study occurred on two different days for the urban and rural school, urine samples from three randomly selected children were additionally collected in the other school than the one where the study took place that day. The latter samples were maintained in a cooled container, after which they were stored at −80 °C and aliquoted later. To avoid any potential impact on downstream analyses, i.e., the investigation of protein biomarkers, no preservative was added to the samples.

#### 2.3.2. Saliva Collection

Several saliva samples per child were collected by the research team. One saliva sample, intended for the investigation of genetic biomarkers, was collected with an Oragene DNA tube (DNA Genotek, Ottawa, ON, Canada). This was only done during the first time point of the field study. A second saliva sample, intended for the investigation of methylation biomarkers, was collected during both time points, with the same type of tube as the first sample. These collection tubes contain reagents to preserve high molecular weight DNA by inhibiting degradation, preventing bacterial growth and therefore maintaining the integrity of the DNA [48]. They allow the samples to be stored at room temperature for a prolonged time (for up to 5 years, according to the manufacturer) until DNA extraction. A third saliva sample was collected at the two time points for the evaluation of epigenetic miRNA biomarkers using an RNA stabilizing container, the Oragene RNA tube (DNA Genotek, Ottawa, ON, Canada). This tube includes an agent ensuring the stability of RNA at room temperature, but only for up to 8 weeks according to the manufacturer. In order to preserve the RNAs for prolonged storage, the tubes were kept on ice during the study and stored at −20 °C at the end of the day. The saliva samples were collected at least 30 min after the start of school and/or after the break to avoid any traces of drinking and/or eating in the samples.

#### 2.3.3. General Health Parameters

In-between the saliva and urine collections, general health parameters, such as the length, the weight and the blood pressure of the child, were measured by the CLB medical staff. Blood pressure was measured by making five consecutive readings using an automated blood pressure instrument (Stabilo Graph, Germany) with a pediatric cuff. The first blood pressure measurement was excluded, as it tends to be inaccurate, and the average of the remaining blood pressure measurements was used for analysis.

#### 2.3.4. Respiratory Health Parameters

FeNO was measured by a trained research team member with the NOBreath monitor (Bedfond, Maidstone, UK). Before starting the FeNO measurements, ambient nitric oxide (NO) was measured to ensure appropriate measuring conditions (with ambient NO preferably <5 parts per billion (ppb) and not more than 35 ppb). Subsequently, at least three measurements were performed, following the guidelines established by the American Thoracic Society [49], with one disposable single-use mouthpiece recommended for each individual. Similar to the collection of saliva, no eating or drinking was allowed just before the measurement. Only after the FeNO measurement could lung function be tested. This was performed by two research team members using the Pocket-Spiro^®^ MPM100 spirometer (MEC R&D, Brussels, Belgium) with a nose clip, following the guidelines [50] and shared throughout the training. However, as children found it difficult to use a nose clip while doing the test, it was repeated without the nose clip. The best of three performed measurements was automatically selected by the software (Patient Data Interface, version 1.1.696), which calculated the forced expiratory volume in 1 s (FEV1), the forced vital capacity (FVC) and the peak expiratory flow (PEF) for each child. In order to gain more insight on potential confounding factors, potentially influencing the measured parameters and biomarkers, research members inquired about the general health state of the child at the time of the study.

### 2.4. Air Pollution Measurement

As mentioned above, the locations of the field study were selected based on their geographic environment, with the aim of targeting different levels of ozone and PM levels expected during distinct periods of summer and winter. Different methods of air pollution measurement were used, i.e., measuring stations and portable monitors.

Days before the start of the field study, the Flemish Environment Agency (VMM, Antwerp, Belgium) installed a measuring station in the parking lot of the rural school and started an air quality measurement campaign, continuously monitoring ozone, PM_2.5_ and PM_10_, black carbon (BC), NO and nitrogen dioxide (NO_2_). Ozone was measured with the Teledyne Api T400 (San Diego, CA, USA) by UV absorption. The PM_2.5_ and PM_10_ levels were detected with an aerosol spectrometer, the Palas Fidas 200 (Karlsruhe, Germany), and the BC levels with an aethalometer (Magee Scientific AE33 (Berkeley, CA, USA)). NO and NO_2_ were measured with a chemiluminescence detector (Thermo Fisher Scientific 42i (Waltham, MA, USA). The hourly pollutant levels were monitored without major technical problems except for the measurement of ozone during the summer phase of the study, due to a technical issue. However, data from another monitoring station with similar equipment, installed in the same region, was made available by the VMM. As ozone is a secondary pollutant that does not vary much regionally, using the data of that nearby station was considered to be a very good proxy for the ozone concentrations at the rural school. Due to lack of space near the urban school, this type of measuring station was not installed in the immediate proximity of the school. However, data from a measuring station that was already installed by Leefmilieu Brussel-BIM (Het Brussels Instituut voor Milieubeheer) 700 m from the school was publicly available and the values of the different pollutants could be retrieved (www.irceline.be, accessed on 11 and 14 September 2018 and on 7 and 10 January 2019).

Mobile devices were also integrated in the study. The Airbeam (Habitatmap, Brooklyn, New York, NY, USA), a wearable air monitor, mapped and graphed in- and outside pollution exposure to PM_10_ and PM_2.5_ in real-time via the AirCasting Android app. The Airbeams were installed at both school sites in each classroom of the participating children and on the playground. Additionally, they were also installed at a streetside location for both schools, more specifically next to the measuring station in the parking lot in the case of the rural school. A portable black carbon monitor MicroAeth AE51 (Aethlabs, San Francisco, CA, USA) monitored the BC level every 5 min and was placed at a streetside location for the urban school. This was done in order to obtain more precise measurements of the measured BC in this street canyon, compared to the monitoring station installed 700 m further.

### 2.5. Sample Analysis

#### 2.5.1. Protein Biomarker

Relevant proteins were investigated using urine samples collected during the different phases of the field study by performing a trypsin digest on 500 µL of urine sample. This was followed by quantification with the MRM method, a previously developed and validated high-throughput method based on a mass spectrometry protocol [19]. It allowed for the simultaneous relative quantification of U-CC16, U-β2M and U-RBP4.

#### 2.5.2. Genetic Biomarker

A commercial kit (prepIT-L2P, DNA Genotek, Ottawa, ON, Canada) was used to extract DNA from each saliva sample, collected with a specific Oragene DNA tube at the first time point of the field study, after which the DNA was stored at −20 °C. The DNA was then analyzed with commercial genotyping assays for the SNP *CC16* G38A polymorphism (rs3741240) and SNP *UGRP1* G112A (rs1368408) as previously described [32].

#### 2.5.3. Epigenetic Biomarkers

In addition to genotyping, the suitability of the collected saliva samples was also assessed for the measurement of relevant epigenetic biomarkers. Three potential candidates were selected, as a proof of concept, to investigate this type of epigenetic biomarker in saliva.

Firstly, the TET-1 methylation level (cg23602092) was investigated. PCR and sequencing primers for the *TET-1* region of interest (at cg23602092) were designed using the Pyromark assay design software (Qiagen, KJ Venlo) (Supplementary Table S1). As proof of concept, six randomly selected saliva samples (three from the rural school and three from the urban school, collected during the first time point of the study) were further processed. The selected workflow was similar to the one used in another methylation study [51,52]. Briefly, the DNA was extracted (as described in Section 2.5.2. Genetic Biomarker) and underwent bisulfite treatment, consisting of converting methylated cytosines into uracil, by using the EZ Methylation Gold kit (Zymo Research, Irvine, CA, USA). This was followed by amplification and pyrosequencing of the selected bisulfite-treated DNA samples with the Pyromark Q48 device (Qiagen, KJ Venlo, The Netherlands). The software quantified the amount of methylated cytosines, providing a methylation percentage of the TET-1 biomarker.

Secondly, the investigation regarding the suitability of saliva for the measurement of miRNA biomarkers was performed using commercial kit saliva samples collected at the two time points from two randomly selected children (one from each school) with the Oragene RNA tube which was stored at −20 °C. The exoRNeasy kit (Qiagen, Hilden, Germany) was used for isolation of the fraction containing extracellular vesicles (EVs) and the subsequent extraction of RNA. To quantify the total RNA and the miRNA content in these EVs, a Qubit™ microRNA Assay and a Qubit HS RNA Assay (Thermo Fisher Scientific, Waltham, MA, USA) were performed. The samples were spiked with 250 fmol of *C. elegans* miR-39 (cel-miR-39) as an exogenous control, for normalization of the expression data [53,54]. MiR-191, characterized by its stable expression in saliva [55], was selected as endogenous control. The presence of specific biomarker miRNAs (miR-222 and miR-146a) was investigated for each investigated saliva sample with a 2-step real-time multiplex commercial qPCR assay, carried out on a CFX 96 Real-Time System (Biorad, Temse, Belgium) following the guidelines of Taqman microRNA assays and using a specific protocol for creating custom reverse transcription and preamplification pools.

## 3. Results and Discussion

### 3.1. Field Study Design

Our study aimed at evaluating the technical and logistic feasibility of a future large-scale integrated study to monitor health risks of air pollution episodes in healthy children. This was done via the conduction of a small-scale pilot field study where the use of appropriate non-invasive samples could be investigated to measure potentially relevant biomarkers at an integrated level for monitoring the impact of PM and ozone on their respiratory health. This would then allow for selection of the most efficient approach in view of technical and logistic feasibility and the most optimal design of future large-scale epidemiological studies. The design of the study included several steps, i.e., targeting the selected vulnerable strata, selecting the appropriate non-invasive samples and methods to measure relevant biomarkers at different levels, and selecting the best study setup.

Firstly, as the goal was to set up a study that was able to investigate a vulnerable stratum of the population, specifically children, it was decided to set the age range from 9 to 11 years old. This age group was set below 11 years old to avoid the confounding effect of the prostate potentially producing the CC16 biomarker in prepuberal boys, which would lead to an overestimation of measured urinary CC16 biomarker levels coming from the lungs [56]. To investigate this target group, several locations, such as schools, scouts or summer camps (with overnight stay) or playground holiday camps (without overnight stay), were initially envisaged. Finally, the school setup was found to be the best option, as the other alternatives were associated with several logistic and practical hurdles (Supplementary Table S2). Firstly, planning a study involving at least two time points including the same children in the context of a multi-phase field study was found to be quite straightforward when targeting them in schools. This is in contrast to a playground holiday camp setup where it would seem more challenging to target the same children over different periods as their attendance is not mandatory and might be dependent on the specific holiday schedule of their parents. In addition, summer camps are commonly not organized in the winter when other pollutant levels are usually expected. A two-point study should then be conducted on two different days in the summer, limiting the chances for contrasting pollutant exposure for these two time points. Secondly, the children are automatically grouped by age in school classes, while summer/playground camps can include groups with a broader age range which is less desirable in this type of study. Thirdly, it is easier to select different schools in rural and urban areas with contrasting pollutant levels, while summer camps are often conducted in rural or woody, i.e., potentially less polluted, environments. Most importantly, when selecting schools, there was the possibility of collaborating with a ‘pupil guidance center’ associated to the school, in Belgium embodied by the CLB. This ensured the presence of a medical staff member on site for the measurement of general health parameters (weight, length, blood pressure). This is in contrast with other types of camps, where the presence of medical staff needs to be organized, probably increasing the costs of such a study. Additionally, the CLB could act as a third party to ensure the anonymity of the children throughout the whole study. Indeed, the CLB served as the perfect intermediary for communication between the researchers, the schools, the children and their parents. Additionally, the anonymity was further ensured by handling all collected samples and questionnaires using a prelabelled coded system throughout the whole study. This collaboration with the CLB was also selected to maximize the willingness of the parents to allow their child to participate. However, choosing schools as the setup to conduct this study also had its drawbacks. For instance, they have prolonged closing periods during the different holidays, especially during the summer, when contrasting high ozone levels are expected. Furthermore, the tight schedule and the multiple school activities (exams, gym sessions, field trips, (lunch) breaks) had to be taken into account when planning and conducting the field study. To avoid interference during important classes, organizing a thematic ‘air pollution day’ in the school on the day of the field study involving activities related to this topic might be of interest.

Secondly, the appropriate samples were defined to measure the selected relevant biomarkers at the different levels (genetic, protein and epigenetic) using recognized and standardized methods. Urine and saliva were chosen as non-invasive samples to measure relevant biomarkers to monitor the effect of air pollution in the respiratory health in children. Indeed, from previous studies and based on our field study setup, we found that, for the monitoring of protein biomarkers, urine was found to be easily collected and stored. Using an MRM method, previously validated [19] and applied in a similar study [23], allowed the simultaneous quantification of U-CC16, a biomarker of lung injury, permeability and respiratory diseases, as well as U-β2M and U-RBP4, two potential adjusters for variations in diuresis and renal handling of urinary proteins. For the genotyping and epigenetic methylation biomarkers, previous studies confirmed that saliva is a good non-invasive source [57,58,59], with DNA of such high quality that it could be used as an alternative to blood DNA in epidemiological studies [48,60,61]. The extracted DNA was analyzed using commercially available qPCR-based genotyping assays to investigate the SNPs *CC16* G38A and *UGRP1* G112A, which were both found to be associated with respiratory health conditions and the development of hypersensitive response [26,27,28,29,30]. Additionally, carriers of the A-allele of the *CC16* SNP G38A were associated with lower circulating CC16 levels and with increased odds of asthma [31]. The salivary DNA was also used to investigate the TET-1 methylation level (cg23602092), a potential biomarker differentially methylated following air pollution exposure [37]. Importantly, when investigating epigenetic biomarkers, their spatiotemporal specificity should be taken into account. Urine does not seem to be suitable as it contains several cell types (i.e., blood cells, epithelial cells and malignant cells), making it difficult to identify the origin of the epigenetic change, which is tissue specific. In general, blood is the preferred sample used for human exposure analyses but it less feasible to collect when investigating children in large-scale studies due to the ethical and other issues mentioned above. Saliva, on the other hand, contains mainly epithelial cells from which buccal DNA can be extracted, potentially making it a suitable source for the measurement of methylation biomarkers of environmental exposure impacting the respiratory tract. In this study, the selected strategy to investigate the TET-1 methylation level was bisulfite conversion followed by pyrosequencing, which are well-known methods that have been used as a gold standard for years in other similar studies [62,63,64]. Recently, a study investigated extracellular vesicles (EVs) as a source of miRNA biomarkers of exposure and effect [65]. EVs, present in saliva, can be an attractive source of biomarkers due to their easy accessibility for isolation, their stability and the ability of their content to be transported from cell to cell [66]. Moreover, extracellular non-coding RNAs are thought to have stable and similar expression profiles throughout different fluids [67]. In this study, miR-222 and miR-146a were investigated, two potential biomarkers that are responsive to PM exposure in adults [39,40,41,42]. The selected methods included commercial kits and assays which were successfully used in previous studies involving other biofluids, such as blood and sweat [68,69]. The results of our study confirmed the rationale of using EVs in saliva as a source for investigating specific extracellular miRNAs as biomarkers of air pollution exposure.

Thirdly, this study focused on the most problematic pollutants (PM_10_, PM_2.5_ and ozone) and aimed to contrast levels by performing the feasibility study at two time points, occurring in summer and winter, and at two locations, i.e., two schools located in an urban and a rural area. Several areas were investigated and selected based on maps showing the yearly averages of the different pollutants [70,71]. The most straightforward approach was to select one school in an urban area (expected to be more ‘exposed’) and one school in a rural area (expected to be less or at least differentially exposed). Moreover, by including two distinct time points occurring in two different seasons (summer and winter), chances to target different levels of ozone and PM were maximized. When selecting these schools, it is also important to take into account all possible additional air pollution emission sources based on their location, as well as on their building design. These aspects could impact the outdoor and indoor pollution inhaled by the children. More information on the profiles of the selected schools for this study can be found in the supplementary data (Supplementary Table S3). As the scope of the study was more focused on technical/logistic feasibility and less on the interpretation of pollutant data, no extensive analysis of the potential additional emission sources was done in this study. However, it is important to take this into account in future larger-scale studies.

### 3.2. Field Study Preparation

All completed documents (DIC, assent (Supplementary Figure S2) and questionnaire) were handled by the CLB. The questionnaires were anonymized by the CLB and returned to the research team before the start of the field study. The questionnaire (10 pages) took around 20 min to complete by the parent(s) of the participating child and inquired about the child’s health, their social background and their in- and out-of-house environment. This allowed for the identification of potential confounding factors that could influence the measured outcomes. However, the retrieval of information from the questionnaires was hampered by several bottlenecks. Firstly, a significant number of questions were left blank or only answered partially, making it difficult to interpret some of the data obtained (Table 1). The main questions that were left blank were mostly related to the occurrence or age of a certain condition or activity from early childhood and the mother’s or father’s activities, such as their smoking status. The reason for leaving parts of the questionnaire blank is unclear. One possibility could be that the 20 min needed to complete the questionnaire was too long for the parents. In a number of questionnaires, several questions in a row were left out blank due to the recto–verso layout of the questionnaire. This problem of partially filling in answers could be avoided by distributing electronic versions of the questionnaire instead of the paper version, requiring a reply to each question before proceeding to the next one. Alternatively, health-related questions might better be addressed by including an oral interview with a doctor, yielding a more accurate response. Ideally, this could be complemented with a health examination from a doctor present on the day of the study, including the assessment of respiratory health by a professional. All this would result in a more accurate description of the child’s health. However, when keeping large-scale epidemiological studies in mind, this would demand too much time, be too costly and would require the presence of a parent during the study. Secondly, information regarding the ethnicity of the child was not asked in the questionnaire, due to ethical reasons. However, this information appeared later on to be a relevant parameter of added value for interpreting the measurement of genetic biomarkers and of lung parameters. The questionnaire was based on a previously established questionnaire in the context of a study with children and respiratory health [46,47]. At that time, no questions regarding ethnicity were included, as they were defined as sensitive questions to which people might not want to answer and which therefore might have been problematic in relation to acceptance by the Ethics Committee. However, we have observed that this approach has recently changed and that questionnaires now do sometimes include this type of question. Therefore, although some of the issues around ethnicity can be sensitive, the collection of this information is very important when analyzing genetic biomarkers as well as respiratory health parameters and should therefore be included in future large-scale studies.

### 3.3. Examinations and Sample Collection

Out of the 36 invited children from the urban school and 56 invited children from the rural school, 19 and 23 children, respectively, eventually agreed to participate in the field study (Table 1). All examinations and sample collections were performed within less than a school day by eight staff members. The study in the urban school, involving 19 children, was conducted in 5 to 6 h for time point 1 and 2, respectively. Although the study in the rural school involved more children (i.e., 23 children), it was performed in only 4 to 5 h for time point 1 and 2, respectively. This might be due to the fact that, at each time point, the study was first conducted in the urban school, after which some small bottlenecks could already be identified and subsequently addressed before the study in the rural school three days later. Additionally, several organizational factors such as better coordination with the school schedule and less time lost when transferring the small groups of children between the classes and examination room (because of closer proximity) could have led to a gain in time in the rural school.

The collection of the non-invasive samples (urine and saliva) was easily accepted by the children and was followed by storage at the appropriate temperatures without any major difficulties (Supplementary Table S4). Although urine sampling is easy, cheap and available in large quantities, it is nevertheless important to take into account the timing of its collection, as first or second morning urine samples are preferred for its downstream processing [72,73]. All children delivered saliva samples, except for one child who had difficulties spitting. An important aspect to take into account is the timing of saliva collection, as no drinking or eating was allowed before sampling, which is impacted by the timing of scheduled breaks. Another aspect to consider with both urine (for protein biomarker analysis) and saliva samples (for miRNA analysis) is to store them at at least −20 °C at the end of the day. This is in contrast with the saliva samples intended for DNA purposes (genotyping and methylation analysis) which can be stored at room temperature for a prolonged period of time.

The measurement of height and weight was done without any issue (Supplementary Table S4). Measuring blood pressure, however, was quite time consuming and specific material, i.e., a pediatric cuff, was needed. The assessment of respiratory health parameters (FeNO and lung function) was the most time-consuming step of the examinations but could be performed successfully for almost every child (Supplementary Table S4). However, important to take into account for the lung function measurement was the need of well-trained staff to use the spirometer and the difficulty of using a nose clip for the child. This is in contrast with the FeNO test, which was easily performed for each child. However, after each school break (including children eating and drinking), valuable time (30 min of waiting) was lost, disabling the possibility of immediately measuring FeNO. Additionally, as mentioned before, a main bottleneck of both lung parameter tests was missing information regarding the ethnicity of the child, which was not retrieved from the questionnaire but which was valuable for the interpretation of the data.

### 3.4. Air Pollution Measurement

During the field study, air pollution was measured using stationary and portable methods. The pollutant levels registered by stationary measuring stations (PM_2.5_, PM_10_, ozone, BC, NO and NO_2_), by the portable BC monitor and by the Airbeams (PM_2.5_, PM_10_) on the day of the study are summarized in Table 2.

The measuring station levels were compared to recently adapted WHO guidelines [3], which state that the 24-h average exposures should not exceed 15 µg/m³ and 45 µg/m^3^ more than 3–4 times per year for PM_2.5_ and PM_10_, respectively. No exceedances occurred, except for the PM_2.5_ value measured in the urban school during the winter (19 µg/m^3^). Nevertheless, distinct differences in pollutants between the two locations and between the two time points were observed with the measuring station. Although it would be interesting to aim for pollutant peaks, expected to lead to larger effects at the level of the measured biomarkers, it is challenging when opting for a study setup in schools. This is due to the extensive planning required beforehand and the short-term previsions of weather forecasts. However, aiming for critical values is not per se required, as other studies have shown that even low pollutant levels also impact measurable indicators [6,23,74].

Although the monitoring setup used in the trailer contains the official (reference) methods for measuring air pollution, it cannot be installed everywhere. Due to the urban environment of the school, characterized by minimal available open space, this type of trailer could not be installed in the immediate proximity of the urban school. The use of publicly available data from a measuring station, installed 700 m further, was used instead and could give a rough estimation of pollutant levels near the school. However, cautious interpretation is needed, as differences in pollutant levels between the location of the trailer and the school might be observed due to their different microenvironment. Especially for NO, NO_2_ and BC, the local microenvironment can result in large local gradients. The measuring station is located next to a canal, but the school in question was located in a street canyon where pollutants tend to disperse less than those emitted in an open area.

To try to circumvent this problem, portable monitors were used. The portable MicroAeth AE51 BC monitor has been shown to be very useful in assessing environmental and personal exposure to BC in epidemiological research [75,76]. This instrument was placed at a streetside location for the urban school, where the BC levels were successfully monitored each day of the field study. The main inconvenience of portable monitors is the limited battery life of maximally 24 h and 8 h for the BC monitor and Airbeam, respectively. Surprisingly, the PM_2.5_ values measured by the mobile Airbeams showed contradicting results compared to the trailer values and trends were not always consistent in the rural school. PM_2.5_ levels were higher at time point 1 than at time point 2, contrasting with the measurements from the measuring station, the latter showing the expected trend of higher PM_2.5_ levels in the winter (time point 2) compared to the summer (time point 1). This discrepancy might be due to the higher measurement uncertainty and variable bias that is often found for low-cost PM sensors [77,78] which occurs with high relative humidity. Typically, the sensors overestimate PM_2.5_ concentrations at high relative humidity (above 80%) and are unreliable for larger particles (PM_10_). A previous study also found that this type of monitor might overestimate low PM concentrations and underestimate higher PM concentrations [77]. Although the discrepancies could perhaps (partially) be explained by the measurement uncertainty observed for these types of sensors, we cannot exclude that other unidentified factors might have also played a role in these unexpected outcomes for the rural area. Therefore, without further technological improvements, it does not seem to be advisable to use the Airbeam on its own for the measurement of absolute PM values and to draw significant conclusions related to the impact on respiratory health. However, this easy-to-use and cheap instrument allowed us to compare values measured between different Airbeams. For instance, except for the measurement of levels during the summer time point in the urban school, the classroom PM_2.5_ levels were lower each time than streetside and on the playground. Airbeams could probably be used for comparing personal exposure values between different locations as long as the relationship with official monitors is thoroughly investigated and one is aware of the impact of high relative humidity on the PM levels of low-cost PM sensors. If needed, these humidity effects can be compensated for [79,80]. As this was not in the scope of this pilot study, we have not included this type of calculation. It would of course be of interest to take them into account in future studies to improve the quality of the measurements when choosing portable instruments, preferably in combination with other validated methods. Moreover, these Airbeams could be used for sensibilizing the population, for facilitating community conversations and the development of strategies to reduce pollution [81].

### 3.5. Sample Analysis at Protein, Genetic and Epigenetic Levels

The goal of this small-scale study, involving a small set of samples, was to assess the technical feasibility of detecting relevant biomarkers at different levels (i.e., protein, genetic and epigenetic) by using the appropriate methods. These biomarkers are sensitive indicators of airway damage and inflammation (protein biomarkers) and of genetic and epigenetic variations. This study did not focus on potential biologically relevant associations between them or with air pollution. The feasibility investigation was conducted on a selection of the collected non-invasive samples and led to successful detection and analysis of relevant biomarkers at each level (protein, genetic and epigenetic). The measurements were performed using validated and/or commercially standardized methods, yielding success rates comparable with other similar studies (Supplementary Table S4).

Based on our field study setup, we found that, for the monitoring of protein biomarkers, urine could be analyzed using a validated MRM method [19]. The use of only 500 µL of urine was more than sufficient for performing a trypsin digest and to deliver a qualitative protein sample for analysis. In this study, we used this MRM method which allowed for the simultaneous relative quantification of the proteins of interest, i.e., CC16 and its adjusters (β2M and RBP4), in almost all urinary samples of this study (Supplementary Table S4). This was especially interesting for the quantification of one of the adjusters, β2M, which tends to degrade at acidic pH in urine. Quantification through classical immunoassays might therefore be challenging but is not the case using this method, as it is based on the quantification of protein fragments. Moreover, this multiplex mass spectrometry method is not limited by inter-assay variations that would potentially occur in the individual simplex immunoassays of each protein, therefore reflecting more accurately the true abundances within the samples. Recently, the use of MRM technology for protein research has increased due to its high sensitivity, high degree of reproducibility and repeatability, and its ability to be used in an automated high-throughput and multiplex setup and over a wide dynamic range (4–5 orders of magnitude). Although the purchase of a mass spectrometer is quite expensive, it can be used for numerous years and applications. Moreover, once validated, as was the case in this study, the MRM method is a time-efficient, cost-efficient and high-throughput way for measuring multiple proteins in a high number of samples.

For the investigation of genetic biomarkers, such as the selected SNPs in this study, the collected saliva, stored at room temperature, was used to extract high quality and quantity DNA. All collected samples were successfully genotyped except one, probably due to a technical issue that occurred during the DNA extraction (Supplementary Table S4). The quality and quantity of the extracted buccal DNA were sufficient to use the selected genotyping qPCR-based assays of *CC16* G38A and *UGRP1* G112A without any interference of contaminating bacterial, fungal or food DNA usually present in saliva. If needed, even urine could be used for genotyping, although this yields inferior results compared to the salivary DNA [33], making it a less preferred biofluid for genetic biomarker investigation unless no other samples are available.

For the investigation of epigenetic biomarkers, differently stored saliva sample were used depending on the type of investigated epigenetic biomarker (miRNA or methylation). Firstly, the saliva samples stored at room temperature were used for investigating the methylation patterns of *TET-1*, a biomarker potentially related to changes in air pollution or asthma. From six randomly selected salivary DNA samples, all were successfully bisulfite converted and pyrosequenced (Supplementary Table S4) resulting in interpretable methylation results, which can be found in the Supplementary data (Supplementary Figure S3). From our results, we can conclude that the use of PCR and pyrosequencing allows individual methylation sites to be assessed with high accuracy. Secondly, the saliva samples stored at −20 °C were used for the detection of two potential miRNA biomarkers and two miRNA adjusters (one internal and one external control) in the extracellular fraction of the samples. EVs from four randomly selected saliva samples were isolated and subsequent miRNA extraction followed by a Qubit analysis showed that total RNA, as well as miRNA, was present in the extracted samples. A multiplex qPCR was performed and each specific miRNA, selected as a potential biomarker (miR-146a, miR-222) or as exogenous (cel-miR-39) or endogenous control (miR-191), yielded detectable signals and Cq values (Supplementary Table S4). The signal of the exogenous control was comparable for each sample, confirming the limited technical variability between the different sample extractions (Supplementary Table S5). The endogenous control could be used to normalize the data in further analysis. The results of our study demonstrate the adequate use of EVs in saliva as a source for investigating specific extracellular miRNAs as biomarkers of air pollution exposure.

### 3.6. Evaluation of Willingness to Participate

The rate of children that participated in this study was slightly higher in the urban school (53%) compared to the rural school (41%), as illustrated in Table 1. A child could only participate in the study if both a parent and the child agreed to it. Out of 17 non-participating children from the urban school, 2 of them actually agreed to participate but their parent did not allow it. Inversely, three parents agreed for their child to participate but the child opposed it. This shows the impact of having to include the choice of both parties to participate or not in this kind of study (inherent to studies where children are involved) on the participation rate.

Feedback on the conducted study, and more information on the motivation or reticence to participate again in such studies, was obtained from follow-up letters completed by parents (of both participating and non-participating children) at the end of the study. A total of 54 letters were returned from the urban and rural school combined. The limited number of follow-up letters (10, of which 9 were from participating children) that were collected from the urban school could be due to a communication error, the limited time (2 weeks) between distribution and collection and/or the lack of sending reminders. This is in contrast to the high response rate (79%) of the rural school for both the parents of participating and non-participating children, where 44 completed letters were returned. As this study involved non-invasive sample collection, a higher participation rate was expected in comparison to similar studies requiring blood samples in children and where similar participation rates around 50% had been observed [82,83]. Based on the data obtained from the follow-up letter from all the participating children, half of the parents (50%) were enthusiastic about their participation. No reticence of the children for giving saliva or urine samples was observed during the study in giving saliva or urine samples. This is confirmed in the follow-up letter, as 93% of the parents would agree to let their child participate again to the same kind of study, including giving the same type of samples. However, only 46% of them would still agree if blood sampling was required. Sharing the personal results or the child’s blood type with the parents at the end of the study had only a limited effect on increasing the acceptance of blood sampling (50% and 46%, respectively).

A main reason for the limited participation rate might be the limited time (2–3 weeks) offered between the distribution and collection of the forms and the fact that no reminder was sent. Additionally, researchers had only limited access to parents. These problems can occur more often in urban schools where parent involvement in the school setting can be more limited, due to language and cultural differences [84,85]. However, in our case, the urban school had a higher participation rate than the rural school. This was perhaps due to the selection of a motivated and actively involved urban school who already participated in a previous air pollution-related study. Additionally, an information session was organized at that school, in the larger context of a monthly gathering, which allowed the parents to ask questions beforehand and to have a better understanding of what the study implicated.

A second reason might be the reticence of the child, which is illustrated Table 1. The majority of the children that did not want to participate were scared (25%), not interested (33%) in the study or did not specify a reason (21%). Other less occurring reasons were the reticence to give a urine sample, medical reasons, the lack of time to get the DIC and questionnaire filled in or that the child was not enrolled at that school at the start of the study.

### 3.7. Ideal Workflow of Larger-Scale Field Study

Although the number of children that finally participated in the study was limited, it allowed for the elaboration of a practical workflow and the identification of bottlenecks and tools for future larger-scale integrated epidemiological studies by including adaptations at different levels of the field study setup.

#### 3.7.1. Adaptations Based on the Workflow of the Feasibility Study

##### Field Study Design

The selection of which adaptations to make in the future study depends on the type of study design, its available resources and budget, and the required sample size. When extrapolating to large-scale studies, power sample size calculations will need to be performed to define the appropriate sample size. The sample size will depend on which biomarkers (genetic, protein, epigenetic) are investigated and the associated expected effect size of such an integrated approach. We would already recommend a study design with at least two time points (two-point study design) with the same subjects to increase power.

##### Field Study Preparation

In our study, the chosen setup led to a reasonable but not extremely high participation rate of the school children. In order to increase the participation rate in future studies, several modifications can be made. First, the timing needed for distributing the documents and for sending reminders should be increased in order to increase the number of returned documents. Secondly, face-to-face recruitment may be more effective and would allow the parents to ask questions. Therefore, information sessions should be organized in each participating school. A researcher should be present to provide further explanations about the study and to invite the parents to agree to their child’s participation. This would implicate more time needed for preparation; however, optionally, parents could immediately agree to the participation of their child and, if the child also agrees, start filling in the requested documents during this info session, leading to a gain in time.

##### Examinations and Sample Collection

In order to conduct the study during school hours, the sampling and examinations were performed on two separate days in the two schools. By increasing the number of staff members present on site, the timing of each study could be reduced. It could potentially allow the field study to be conducted on the same days in several schools or sites simultaneously. When extrapolating to a larger-scale epidemiological study, a significant increase in the number of sites, the number of time points and the number of children would give additional information on the dynamic effect of air pollution. However, this setup presents certain challenges as it requires supplementary measuring instruments (spirometer and FeNO instrument), more staff and more time. Alternatively, to reduce the additional work of the research staff at school during the study, some of the sampling and examinations (weight, height, blood pressure, saliva sampling intended for genotyping) could be done by the CLB at the yearly consultation. Urine sampling can be done by the research staff coming on site or by the teachers, if they agree to it. Alternatively, self-sampling could be envisaged (see further in Section 3.7.2). The storage conditions of the non-invasive samples are an important parameter to consider for the feasibility of a larger-scale field study. Urine samples should be stored frozen, at a temperature of at least −20 °C. Although proper storage cannot be ensured, it should not be problematic in a school, where there is usually access to a freezer. The saliva collection, intended for genotyping, is only required once, whenever convenient, and can be stored at room temperature for a prolonged time. In cases where the epigenetic biomarker measurements are included in the workflow, additional saliva sampling at different time points can easily be included, especially for methylation investigation where the saliva samples can stay at room temperature.

Contrary to the non-invasive sampling and consecutive measurement of U-CC16 and SNP CC16 G38A, conducting FeNO and spirometry tests in the context of a larger-scale study involving multiple locations and time points becomes challenging. Indeed, although the FeNO measurement is easy, the spirometry test can be challenging to perform in children, and both tests are quite time-consuming, necessitating trained staff and multiple instruments. This significantly increases the costs and the logistic complexity. This is in contrast with collecting urine and saliva in children, which is quite straightforward and cheap, even for multiple locations and time points. Moreover, when no saliva is available, for instance in older/historical collections, genotyping can even be done with DNA extracted from urine instead of saliva, although a lower, but still acceptable, success rate might be expected [32]. The qPCR-based genotyping can easily be upscaled. The high-throughput MRM method can be used to measure U-CC16 in numerous samples. Furthermore, U-CC16 gives information regarding the integrity of the epithelial barrier of the deep lung, which is a main target of air pollution toxicants, making it a sensitive biomarker. Of course, as for all instruments that are needed for specific measurements, this requires an additional investment but they can be used for numerous years and applications. Moreover, the main advantage of both methods is that, besides being used for analyzing U-CC16 and the SNP G38A, they also allow for the inclusion of more potential current and future biomarkers and can even be used to measure samples retrospectively, in contrast with FeNO and lung function measurement. Therefore, in cases where it is challenging to measure FeNO and lung function in specific larger-scale settings, it might be more feasible to focus on the more straightforward collection of saliva and urine and the measurements of U-CC16 and the SNP G38A. This integrated approach could serve as a potential proxy for the FeNO measurement, as both parameters indicate a possible increase in inflammation of the lungs [86,87,88]; however, U-CC16 would also additionally indicate early adverse effects and injury on the lung.

##### Air Pollution Measurement

The continuous monitoring stations can determine ambient exposure levels with a high degree of temporal accuracy and precision. However, when increasing the number of sites, this approach could show limited spatial coverage in some areas. Indeed, these monitoring stations can be quite voluminous, therefore challenging the installation in each desired location. Furthermore, their installation, maintenance and calibration are quite time demanding and costly. Alternatively, the use of spatial temporal interpolation methods which model daily residential exposure levels could provide the necessary information about pollutant levels at the child’s home and/or school address in future large-scale studies, although this would require additional resources for analyses. These types of models are especially useful for the measurement of ‘regional’ pollutants such as ozone and PM_2.5_ but less applicable for ‘local’ pollutants such as BC and NO_2_. Indeed, although high resolution Kriging regression models for BC and NO_2_ exist, the quality and uncertainty of the model data is very dependent on the input data (e.g., traffic data). Especially for smaller streets, good traffic data is often not available or is dated. Additionally, these models are usually only validated for longer periods of time. In Belgium, these are usually based on yearly averages and occasionally on monthly averages. When accurate data is required in the context of quantitative studies, for relatively short time periods (e.g., 24 h) it is preferable to work with measuring stations where possible. If insufficient budget is available, complementary information can be obtained with the use of portable sensors, which are quite cheap and easy to use and can be used in multiple locations simultaneously. Including these types of non-regulatory monitors to complement a stationary setup can increase the accuracy of highly variable pollution exposure measurements, especially in an urban setting. However, despite the availability of a number of commercial wearable monitors, they often show a size and sensitivity trade-off due to their limited technologies. Cautious interpretation is needed, as there can be large variability in performance between different types of instruments, as well as within the same type of instrument over time [89,90]. Further investigation (or even future development of this technology) is needed to select the most suitable instruments.

##### Sample Analysis (Genetic, Protein, Epigenetic)

The methods selected in this feasibility study, focusing on a limited set of biomarkers, allowed for the measurement of one protein biomarker, two genetic biomarkers and three epigenetic biomarkers that have been reported before to be associated with respiratory health or air pollution exposure. This was an example of a combination of different types of biomarkers being measured simultaneously, giving integrated insights on the health effects of air pollution exposure in children. Importantly, using the described workflow, additional biomarkers for air pollution exposure and respiratory health, or biomarkers for other exposures and diseases, could be included. Indeed, using the MRM method allows for the measurement of numerous current or future protein biomarkers which could be added in the analysis without substantially increasing the analysis costs. However, prior knowledge of these target proteins is needed to include them in the method. Additionally, sometimes technical or biological challenges, inherent to a candidate protein, can lead to unsuccessful measurements [19].

Our study, as well as previous studies, showed the importance of integrating protein outcome with genetic background in future large-scale studies. In this study, the genetic information was obtained for two specific SNPs using salivary DNA without any unfavorable effects of non-human DNA possibly interfering in qPCR-based genotyping assays. These assays could easily be upscaled to analyze more samples and decrease the cost per sample even more. Nevertheless, in a more high-throughput context, such as whole genome sequencing which allows for the screening of a large variety of additional SNPs, the non-human contaminating DNA derived from saliva can confound the outcome and might result in the identification of false variants not present in the human genome [91]. DNA derived from blood samples is usually recommended for WGS. However, other high-throughput molecular genotyping platforms such as arrays exist to target multiple SNPs and still use saliva as a non-invasive source [92]. These arrays allow for the investigation of a larger spectrum of genetic biomarkers to be conducted in future large-scale studies if other proteins need to be included in the integrated approach. Moreover, the investigation of the impact of genomic variants in specific groups with specific genomic risks can result in more effective and cost-efficient monitoring and better prevention strategies [93,94]. The balance between increased cost for such high-throughput technologies, compared to cost-efficient qPCR-based methods, should be evaluated in view of the added value of increasing the number of SNP biomarkers and obtaining additional information for exposure monitoring.

Finally, saliva or urine can also both be used as a non-invasive source to investigate epigenetic biomarkers of different types of exposures or diseases [57,95,96], albeit taking into account the cell-type specificity of the epigenetic changes. If a choice should be made, based on limited resources and budget, perhaps the investigation of epigenetic biomarkers should be excluded from the workflow, as additional methods are indeed required to measure them. In this study, saliva was used to perform a PCR and pyrosequencing, allowing individual methylation sites to be assessed with high accuracy. If more methylation biomarkers were to be targeted, higher DNA input would be required and using pyrosequencing might be challenging, as a multiplex setup, while possible, is quite laborious to design [97,98]. Nevertheless, a number of alternative methods exist, including whole genome methylation profiling or digestion based assays followed by PCR or qPCR [99]. Again, this will increase the cost of the analysis and the added value of increasing the methylation biomarkers to be measured should be carefully evaluated. Similarly, if more miRNA biomarkers were to be targeted in salivary EVs, this selected method, starting with the same saliva amount, can theoretically target up to 96 potential miRNA candidates using a pool of individual TaqMan miRNA assays. However, at this level, it would be quite time consuming and prone to human error. Therefore, other alternatives such as microarrays could be considered [100].

With increasing knowledge of the genome, proteome and epigenome, it becomes clear that the isolated analysis of different levels of biomarkers only provides a linear view of a multidimensional landscape. Ideally, a study should include an integrated investigation combining proteinaceous, genetic and/or epigenetic levels. In recent years, a rapid increase has been observed in the number of studies in which integration was successfully applied, as well as in the number of tools developed to facilitate the integration [23,101,102,103,104]. Such an approach optimizes the use of potential health biomarkers in epidemiological studies to reach an in-depth understanding of interrelated or complementary relationships of the biomarkers and of certain disorders as well as to identify potential risk factors.

#### 3.7.2. Alternative Workflow: Self-Sampling

The described workflow is quite flexible and allows for the modification of the number and the type of biomarkers, as well as the type of exposure and target group. Therefore, depending on the main goal of the future larger-scale study, the selected biomarker, target group and some of the parameters or other steps of the workflow described above can be modified or removed from the proposed design, resulting in alternative workflows.

Schools are often solicited for all types of studies, requesting additional time and efforts from them, which could have a negative impact on their motivation to participate and which exceeds the typical responsibilities of teachers. Moreover, not all countries have schools collaborating with a CLB to serve as a contact point between the research team and the participant to ensure the anonymity of the children and to perform a part of the measurements. When this center is not available, other options should be envisaged to take over these tasks. Another third party other than the CLB needs to be identified to ensure anonymity and to be the contact point between the participant and the researcher. In a similar way to the CLB, this third party would need to work with codes to ensure anonymity and distribute all necessary documents (DIC, questionnaires) in order to obtain personal and medical information. This could potentially be conducted by a member of school staff; however, if not available, an online approach could be set up which would simplify the analysis and reduce the amount of unanswered questions in the questionnaire.

The required sample collections could be performed by additional staff members from the research group, but this will indeed increase the costs and manpower needed for conducting this type of study. Alternatively, self-sampling could be envisaged. This includes sampling of saliva and urine at the participant’s home or school where they could be stored at room temperature and/or at −20 °C, depending on its downstream application. Self-sampling removes the context of working in schools from the workflow and allows for the collection of samples in other countries or other types of location than regular schools. It also provides the possibility of collecting more samples at more time points. The sampling rounds could be organized more in accordance with the weather and air pollutant previsions. From our study, we could conclude that it was very challenging to plan a whole field study taking into account the weather previsions and that it is therefore almost impossible to conduct it during a pollution peak. Although effects have also been observed when pollutant levels were low, it still seems more insightful to conduct the sample collections when higher pollutant levels are observed, as the effect on the measured biomarkers is expected to be more substantial. Self-sampling would facilitate this setup for sampling at moments when high pollutant levels are expected.

This workflow, based on self-sampling, does not include the measurement of general and respiratory health parameters anymore. Depending on the goal of the study, some of these measurements could be removed from the workflow or, alternatively, be measured by additional research staff members, taking into account additional costs and resources. However, this would remain challenging in a multiple sampling and time point setup. Weight and height could be measured by the parents at home and be proxy reported by them. However, cautious interpretation is needed as inconsistencies can occur due to the use of different instruments and different operators. Moreover, home-measured weight and height can show poor accuracy compared to direct measuring [105], although its impact on the analysis of the measured outcome in the specific context of this study has not been investigated yet. Alternatively, the CLB (if available) or the parents could provide data from the latest medical appointment of their child. Respiratory health parameters (FeNO and lung function) cannot be measured by the research team anymore. However, as mentioned above, their measurement requires trained staff and multiple instruments which increases costs and logistic challenges, and the measurement of lung function is quite challenging to perform in children. Therefore, in the case of larger-scale studies where self-sampling is envisaged, limiting the measurements in the workflow to the integrated analysis of U-CC16, described as a potential marker of early lung damage, as well as the SNP G38A with the described methods could be considered, allowing for the measurement of numerous samples with high-throughput and even providing the possibility of including more potential biomarkers when needed.

Opting for self-sampling should be combined with personal exposure information. This can be obtained through data from measuring stations, if available, or, more easily, through modelling data. Ideally this would be combined with self-monitoring of air pollution exposure through the use of performant portable sensors. After an explanation at the start of the study, saliva and urine could be collected from children at one or multiple time points in the schools or at home. This would provide a dynamic view on the self-exposure of the child, not only in school but also at home and during transport.

Self-sampling also allows this type of study to be performed in other strata of the population, such as elderly people, where collection of blood might also be technically more difficult. When these other vulnerable strata of the population would be envisaged, adaptations would of course be needed, but part of the design could remain the same. This would imply changes at the preparation level of the study, but less regarding the measurement methods for the selected biomarkers and for air pollution monitoring. When envisaging this type of study in nursing homes for instance, as with schools, the subjects could be followed-up with easily over time, sampling should not be problematic and medical staff would be present for potential additional examinations. The choice of biomarkers could remain the same but could also be adapted when using the described flexible measuring methods. Indeed, our study involved the investigation of respiratory health, but we believe that this design could also be applied to other health topics and adverse effects, albeit with some adaptations of course at the level of biomarker selection and exposure measurement.

## 4. Conclusions

This small-scale field study evaluated the technical and logistic feasibility of investigating the effect of air pollution on the health of children and to extrapolate it to future larger-scale settings using an integrated approach. The proposed approach took into account the strengths and the limiting factors related to conducting this type of study specifically in children, the collection and storage of non-invasive samples, and the selected biomarker and air pollution measurement methods. The use of urine and saliva as a source of biomarkers is a valuable tool in the context of larger-scale epidemiological studies where blood collection, especially in children, is generally less accepted. The use of specific cost-efficient recognized methods could allow for the simultaneous measurement in multiple samples of multiple relevant protein, genetic and epigenetic biomarkers. It also allows the for possibility of extension to more future interesting biomarkers that can even be measured retrospectively once their detection is validated and the non-invasive samples are collected. Using this integrated approach optimizes the use of potential health biomarkers by providing insight on interrelated or complementary relationships and roles of various types of molecules in cells of an organism. The specific protocol in this study will allow for the assessment of potential risks and impacts of air pollution exposure on the respiratory health of children through monitoring, which is critical for the development of policies or measures for these pollutants at a population level. Moreover, future studies could also use a similar workflow to investigate the effect of air pollution on other vulnerable strata of the population, albeit with some adaptations at certain levels of the design.

## Figures and Tables

**Table 1:** Overview of field study participation rate and information retrieved from questionnaire and follow-up letter.

	Urban			Rural			Total		
Participation ^a^	*n*	%	N	*n*	%	N	*n*	%	N
Children participating	19	53	36	23	41	56	42	46	92
No response/no interest	17	47	36	33	59	56	50	54	92
Parents accept but child does not want to participate	3	18	17	1	0	33	4	8	50
Child accepts but parents do not want child to participate	2	12	17	0	0	33	2	4	50
Both parent and child do not want to participate	12	71	17	32	97	33	44	88	50
**Questionnaire**									
Completed questionnaires (partially or fully completed)	19	100	19	23	100	23	42	100	42
Type of questions answered incompletely ^1^									
Frequency or age of a certain condition in childhood	4	53	7 ^3^	5	68	7 ^3^	9	60	14 ^3^
Frequency of a certain activity performed by the child	3	43	9 ^3^	4	59	9 ^3^	7	39	18 ^3^
Questions related to smoking habits of the mother or father	8	53	13 ^3^	1	33	3 ^3^	9	56	16 ^3^
Incomplete probably due to recto–verso layout	2	11	19	0	0	23	2	5	42
**Information retrieved from returned follow-up letters**									
**Completion**									
Completed by parents of all children (participating and non-participating)	10	28	36	44	79	56	54	59	92
Consent received from child	9	90	10	21	48	44	30	56	54
Consent received from parents	9	90	10	23	52	44	32	59	54
Completed by parents of participating children	9	47	19	21	91	23	30	71	42
Completed by parents of non-participating children	1	6	17	23	70	33	24	48	50
**Reasons for non-participation in current study**									
Reason for no consent/no participation from the child									
No answer (not filled in)	0	0	1	5	22	23	5	21	24
Scared	0	0	1	6	26	23	6	25	24
No interest	0	0	1	8	35	23	8	33	24
Other reasons ^2^	1	100	1	4	17	23	5	21	24
Reason for no consent for child to participate from the parent									
No answer (not filled in)	0	0	1	7	33	21	7	32	22
Scared	0	0	1	1	5	21	1	5	22
No interest	0	0	1	4	19	21	4	18	22
Only one of the parents consented	0	0	1	1	5	21	1	5	22
Other reasons ^2^	1	100	1	8	38	21	9	41	22
**Feedback from a parent after the study for those who participated**									
No answer (not filled in)	0	0	9	1	5	21	1	3	30
Enthusiastic	3	33	9	12	57	21	15	50	30
Uncomfortable	0	0	9	0	0	21	0	0	30
No opinion	4	44	9	8	38	21	12	40	30
Other/not specified	2	22	9	0	0	21	2	7	30
**Consent for future studies**									
From the children participating in the current study									
Parent not willing to let child participate again	1	11	9	1	5	21	2	7	30
Parents willing give consent again for a new study	8	89	9	20	95	21	28	93	30
Number of times/year that new study can be conducted									
No answer (not filled in)	1	13	8	3	15	20	4	14	28
2×/year	3	38	8	9	45	20	12	43	28
3×/year	1	13	8	3	15	20	4	14	28
4×/year	2	25	8	3	15	20	5	18	28
As much as needed	1	13	8	2	10	20	3	11	28
Consent for sampling	7	88	8	20	100	20	27	96	28
Consent for sample type—not specified (not filled in)	1	14	7	1	5	20	2	7	27
Consent for sample of urine	6	86	7	19	100	19	25	96	26
Consent for sample of saliva	6	86	7	18	95	19	24	92	26
Consent for sample of NALF	5	71	7	9	47	19	14	54	26
Consent for sample of feces	3	43	7	10	53	19	13	50	26
Consent for sample of blood	3	43	7	9	47	19	12	46	26
Consent for sample of blood if parents receive:									
blood type information for their child	0	0	7	13	68	19	13	50	26
personal results from the study for their child	0	0	7	12	63	19	12	46	26
From the children not participating in the current study									
Parent not willing to let child participate again	0	0	1	13	57	23	13	54	24
Parents did not answer (not filled in)	0	0	1	8	35	23	8	33	24
Parents willing to give consent again for a new study	1	100	1	2	9	23	3	13	24
Number of times/year that new study can be conducted									
no answer (not filled in)	1	100	1	2	100	2	3	100	3

N: total number of subjects evaluated for specific statement/category mentioned in first column; *n*: number of subjects that complied with statement/category mentioned in first column; ^a^ data obtained from DIC and assent for parents and child, respectively; ^1^: determined as the number of questionnaires where more than 20% of the parents did not answer a specific question. Average of all the questions related to the same topic. ^2^: other reasons—other activities after school, reticent to give urine sample, medical reason, document not returned in time, child not yet enrolled at that school at the start of the study; ^3^: this N does not correspond to the total number of questionnaires/subjects but refers to the number of questionnaires in which parents answered a main question but did not always give a complete answer to the additional related questions. The number of questionnaires where this type of additional question remained unanswered is represented by *n*.

**Table 2:** Air pollutants, measured at the two time points of the field study.

	Urban		Rural	
Measuring Station	t1 in Summer	t2 in Winter	t1 in Summer	t2 in Winter
Ozone	68	34	80	50
BC	0.7	NA	0.9	2.3
NO_2_	41	49	29	62
PM_10_	13	29	17	24
PM_2.5_	5	19	9	13
**Portable BC monitor**				
Streetside	1.9	5.3	/	/
**Portable Airbeam (PM_2.5_)**				
Streetside	7.1	34.8	22.3	8.9 *
Playground	4	30.4	26.2	8.9 *
Classroom	5.2	12.7	14.6	3.9 *

All pollutants are expressed in µg/m³. For the measuring station, levels from day of the study (lag 0) are shown—Ozone: daily highest 8-h mean ozone concentrations (00h00 till 24h00); PM_2.5_, PM_10_ and BC: daily mean concentrations (00h00 till 24h00). * unexpected low values, potentially due to an Airbeam issue. NO_2_: daily highest mean NO_2_ concentrations (00h00 till 24h00). The pollutant levels measured by the stationary measuring station up to two days (lag 2, lag 1) before the examinations are not shown in the table. For the portable instruments (portable BC monitor and Airbeam), mean concentration of values was measured during the time lapse of the study. PM_10_ and PM_1_ levels measured by the Airbeam are not shown. BC: black carbon; PM_10_: particulate matter with a diameter smaller than 10 μm; PM_2.5_: particulate matter with a diameter smaller than 2.5 μm; NA: not available; /: not measured; t1: time point 1 (summer); t2: time point 2 (winter).

## Data Availability

Not applicable.

## Supplementary Materials

The following supporting information can be downloaded at: https://www.mdpi.com/article/10.3390/ijerph19148531/s1, Figure S1: Detailed overview of test phase, field study and encountered bottlenecks; Figure S2: Graphical simplified document of informed consent (assent) to be signed by the children before agreeing to participate in the field study; Table S1: PCR and sequencing primers for TET1 methylation; Table S2: Comparison of different factors affecting choice of monitoring site; Table S3: Profile and microenvironment of the urban and rural school and possible surrounding emission sources; Table S4: Summary of successful sampling, measurements and analyses of the different biomarkers; Figure S3: TET1 methylation (%) of 6 randomly chosen salivary DNA samples; Table S5: Cq-values (SD) representing the expression of miR-222 miR-146a, miR-191 (endogenous control) and cel-miR-39 (exogenous control).
