# Supplementary material for: Assessment of the Feasibility of a Future Integrated Larger-Scale Epidemiological Study to Evaluate Health Risks of Air Pollution Episodes in Children
Source: Int J Environ Res Public Health. 2022 Jul 12;19(14):8531. doi: 10.3390/ijerph19148531 (PMC9323067; doi:10.3390/ijerph19148531)
Supplement: Supplementary file 1 [file ijerph-19-08531-s001.zip › ijerph-1802279-supplementary.pdf]

Supplementary files

A Test phase

Phase	Duration	Target parameters	Tasks research team/CLB
Design	*2 months*	Vulnerable strata - Children<br>9 – 11 years old<br>Primary school	Drafting DIC, questionnaires, study protocol (research team)<br>Submission to Ethical Committee (research team) [1]
Preparation	*1 month*	Selection of one participating school/CLB<br>Exact location/time point not yet relevant (air pollution not yet measured) [1]	Info session school/CLB/parents [13]<br>Send consent forms/questionnaire to parents/children (through CLB)<br>Reception anonymized list and questionnaires participants (CLB) [18]<br>Installation of material in dedicated room (on the day of the study) [14]
Field study	*1 day*		During school hours in dedicated room and bath room (school) [17]<br>Staff: [19]<br>• 5 researchers<br>• 2 medical staff of CLB
Analysi	*1 month*		Participation rate [16]<br>Identification of first set bottlenecks, to take into account for field study

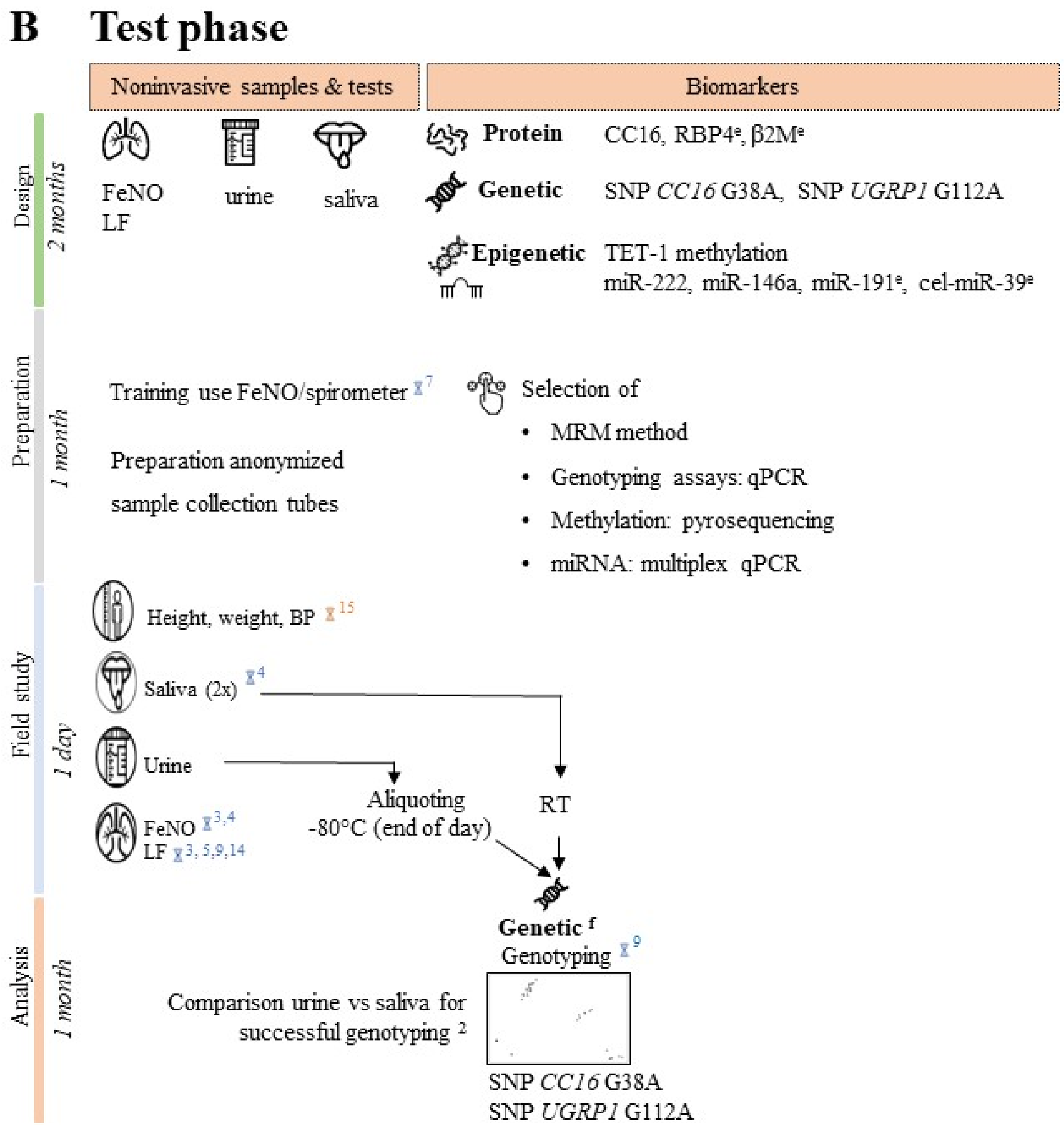
B Test phase
Noninvasive samples & tests
Biomarkers
FeNO
LF
urine
saliva
Protein CC16, RBP4e, β2Me
Genetic SNP CC16 G38A, SNP UGRP1 G112A
Epigenetic TET-1 methylation
miR-222, miR-146a, miR-191e, cel-miR-39e
Design
2 months
Preparation
1 month
Training use FeNO/spirometer 7
Preparation anonymized
sample collection tubes
Selection of
• MRM method
• Genotyping assays: qPCR
• Methylation: pyrosequencing
• miRNA: multiplex qPCR
Field study
1 day
Height, weight, BP 15
Saliva (2x) 4
Urine
FeNO 3,4
LF 3, 5,9,14
Aliquoting
-80°C (end of day)
RT
Analysis
1 month
Genetic f
Genotyping 9
Comparison urine vs saliva for
successful genotyping 2
SNP CC16 G38A
SNP UGRP1 G112A

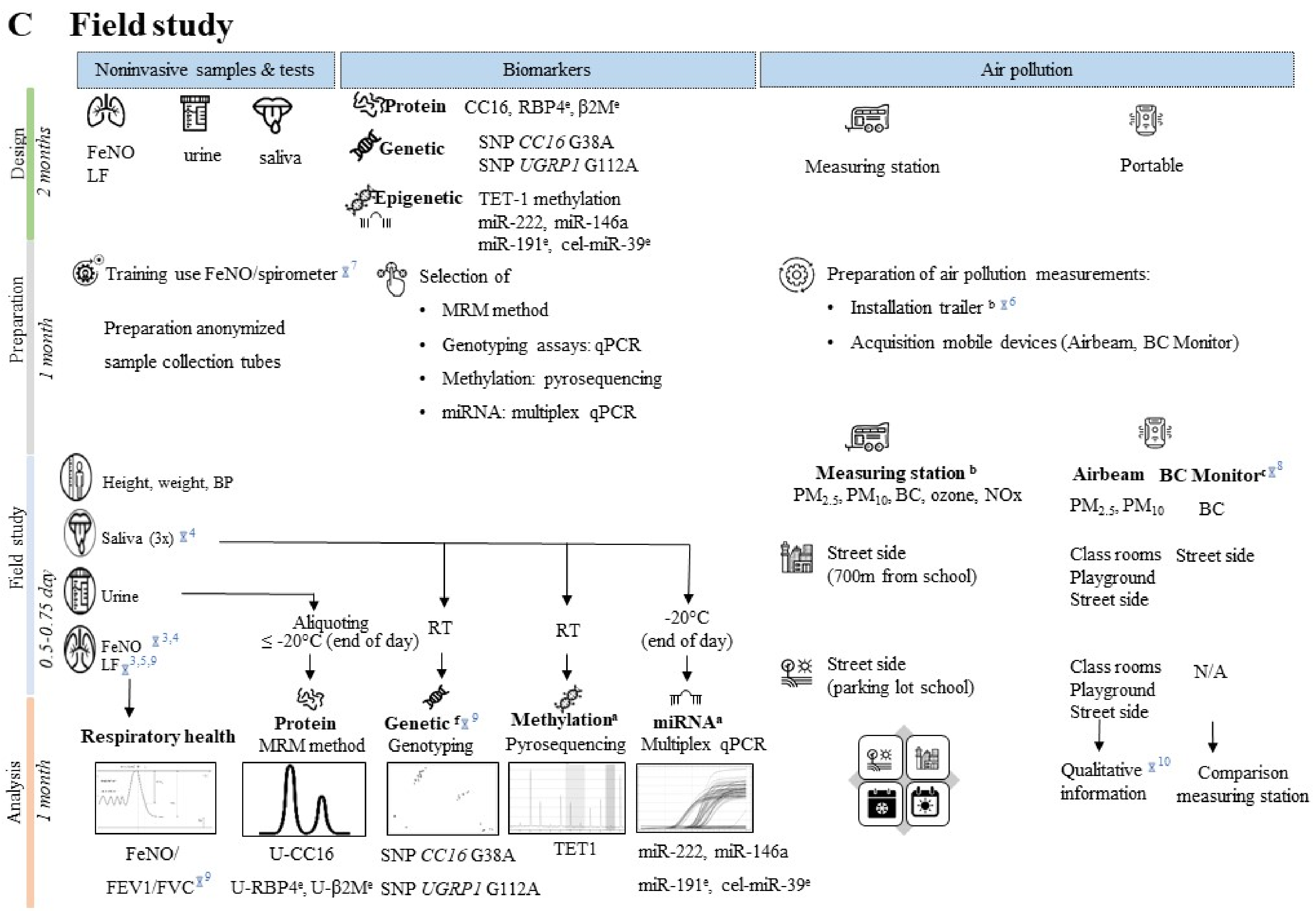

C Field study
Noninvasive samples & tests
Biomarkers
Air pollution
Design
2 months
FeNO
LF
urine
saliva
Protein CC16, RBP4ᵉ, β2Mᵉ
Genetic SNP CC16 G38A
SNP UGRP1 G112A
Epigenetic TET-1 methylation
miR-222, miR-146a
miR-191ᵉ, cel-miR-39ᵉ
Measuring station
Portable
Preparation
1 month
Training use FeNO/spirometer 7
Preparation anonymized
sample collection tubes
Selection of
• MRM method
• Genotyping assays: qPCR
• Methylation: pyrosequencing
• miRNA: multiplex qPCR
Preparation of air pollution measurements:
• Installation trailer b 6
• Acquisition mobile devices (Airbeam, BC Monitor)
Field study
0.5-0.75 day
Height, weight, BP
Saliva (3x) 4
Urine
FeNO 3,4
LF 3,5,9
Measuring station b
PM2.5, PM10, BC, ozone, NOx
Airbeam
BC Monitor c 8
PM2.5, PM10
BC
Street side
(700m from school)
Class rooms
Playground
Street side
Street side
Aliquoting
≤ -20°C (end of day)
RT
RT
-20°C
(end of day)
Street side
(parking lot school)
Class rooms
Playground
Street side
N/A
Analysis
1 month
Respiratory health
Protein
MRM method
Genetic f 9
Genotyping
Methylationᵃ
Pyrosequencing
miRNAᵃ
Multiplex qPCR
Qualitative 10
information
Comparison
measuring station
FeNO/
FEV1/FVC 9
U-CC16
U-RBP4ᵉ, U-β2Mᵉ
SNP CC16 G38A
SNP UGRP1 G112A
TET1
miR-222, miR-146a
miR-191ᵉ, cel-miR-39ᵉ

D Field study

Phase	Duration	Target parameters	Tasks research team/CLB
Design	*2 months*	Vulnerable strata - Children<br>9 – 11 years old<br>Primary school	Drafting DIC, questionnaires, study protocol (research team)<br>Submission to Ethical Committee (research team)[1]
Preparation	*1 month*	Selection of 2 schools/CLB<br>Rural Urban	Info session CLB (not school/parents)<br>Send consent forms/questionnaire to parents/children (through CLB)<br>Reception anonymized list and questionnaires participants (CLB)[11]<br>Installation of material in dedicated room (1 day before the study)
Field study	*0.5-0.75 day*	Conducted on 2 time points[1]<br>Winter Summer	During school hours in class room and bath room (school)<br>Staff:<br>• 6 + 1ª researchers<br>• 2 medical staff of CLB<br>Follow-up letter for parents[11, 12]
Analysis	*1 month*		Participation rate<br>[2,9,11] Analysis questionnaire & follow-up letter:<br>• Medical history/ confounding effects<br>• Willingness to participate again/give (other type of) samples

➔ Extrapolation to large scale studies/other conditions and affections

E Bottlenecks

[1] Timing: take into account holiday periods/tight schedule of school. Not possible to perform the study in the two schools on the same day due to schedule, number of tests and examinations and limited staff

[2] Significant amount of data missing due to (1) questions not completed, (2) limited number of forms collected

[3] Time consuming

[4] No food or drink 30 minutes before test/sampling

[5] Difficult to perform in children

[6] Difficult to install in every location, especially urban setting with limited open space available

[7] Extensive training needed for its use

[8] Limited battery life

[9] No information available on ethnicity of child

[10] Technical characteristics disable the use for absolute pollutant values, only qualitative information available

[11] Limited time between distribution and collection of the documents

[12] Limited number of forms collected in urban school

[13] limited enthusiasm of participating school/CLB → field study: select motivated school and/or organize additional info session, including for the parents to increase willingness to contribute and participate to this study.

[14] Much time : lost due to installation material in class room on the day of study→ field study: install all equipment at least one day before study

[15] BP measurement not successful due to technical issue → field study: need of specific pediatric arm cuff

[16] Participation rate is 65 %. Reasons for (non-) motivation to participate? → field study: collect more information by distributing follow-up letter

[17] Not practical to perform sampling and measurements in class room, interferes with class and sport lessons and other activities (tests) → field study: conduct study in separate dedicated room. Increase communication with school to avoid tests and sport lessons during day of field study

[18] Limited time between distribution and collection of the documents. Reception of documents even on the day of the study (loss of time) → field study: increase timing between distribution and reception of documents. Avoid reception of documents on the day of the study

[19] CLB workload too high on the day of the field study → field study: limit the workload of the CLB to the minimum. Increase the researchers staff.

Figure S1: Detailed overview of the test phase (panels A and B), the field study (panels C and D) and the encountered bottlenecks (panel E). Description of the design, the preparation and the conduction of the field study and its test phase. The different tasks of the field study were conducted by members of the research team and medical staff members of the CLB. Noninvasive tests were performed, including the measurement of height, weight, blood pressure, spirometry and FeNO. Noninvasive samples (saliva and urine) were collected in which biomarkers were measured at different levels (protein, genetic and epigenetic), using cost-effective high throughput methods. The air pollution exposure at the school was measured, using stationary and portable methods.

[1] The study protocol, questionnaires, DIC of the test phase (see Figure S1 (B)) were slightly modified after the test phase. An addendum was submitted to and accepted by the Ethical Committee before the start of the field study.

[2] S. J. D. Nauwelaerts et al., "Selection of a noninvasive source of human DNA envisaging genotyping assays in epidemiological studies: Urine or saliva?," J Biomol Tech, p. jbt.20-3101-004, Apr. 2020, doi: 10.7171/jbt.20-3101-004.

[a] : on selection of samples

[b] : only in rural school

[c] : only in urban school

[d] : additional urine samples collected for 3 children on day of field study in other school by additional staff member of the R&D team

[e] : measured to adjust

[f] : DNA extracted only on saliva samples collected during first time point of the field study

[g] : one extra person of the research team needed in the other school for collecting the additional urine samples

β2M: beta-2-microbglobulin; BC: black carbon, BP: blood pressure, CC16: Club cell protein; CLB: Pupil guidance center; DIC: Document of informed consent; FeNO: fractional exhaled nitric oxide; FVC: Forced vital capacity; FEV1: amount of air you can force from your lungs in one second; miR: micro RNA; MRM: multiple reaction monitoring; N/A: not applicable; NOx: nitrogen oxides; PM: particulate matter; $PM_{2.5}$: PM of diameter less than 2,5 micron; PM_{10}: PM of diameter less than 10 micron; RBP4: retinol binding protein 4; RT: room temperature; SNP: single nucleotide polymorphism; TET-1: Ten-eleven translocation methylcytosine dioxygenase 1; U-CC16: urinary CC16; U-β2M: urinary β2M; U-RBP4: urinary RBP4; UGRP1: Uteroglobin related protein 1

Created using icons from the Noun Project (see Supplementary file S7 for more details)

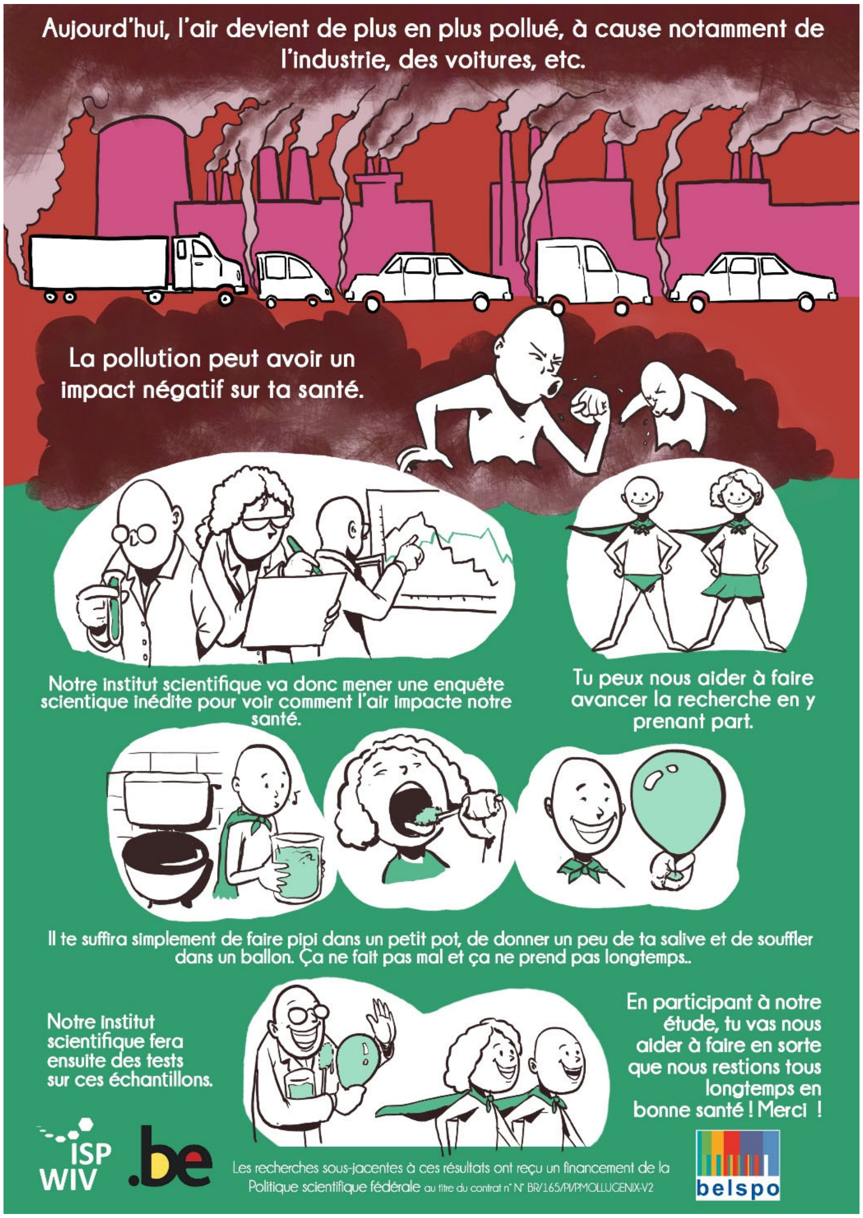

Figure S2: Graphical simplified document of informed consent (assent) to be signed by the children before accepting to participate to the field study. Figure made by J. Van Hautte.

Table S1: PCR and sequencing primers for TET1 methylation

Primer	Sequence (5' → 3')
Forward	TGTGTTATGATTGTTGGAAGAAGTA
Reverse	BIOTIN-ATTCCCTAACTCTAAACTAAATATCTACAA
Sequencing	TTTTTATTTTTTTATTTATAAAGTG

Table S2: Comparison of different factors affecting choice of monitoring site.

Determining factors	(1) school	(2) summer/scouts camps (with overnight stay)	(3) playground camps (without overnight stay)
same children over different time points with different exposure	Yes, mandatory presence	Not always same children participating to each (yearly) camp, might depend on parents' holiday schedule Only in the summer (limited exposure differences)	Not always same children participating, might depend on parents' holiday schedule
different locations with contrasting pollutants	yes (urban and rural)	usually rural or woody	yes (can be urban or rural)
easy collaboration with medical staff	yes, due to collaboration with CLB (in Belgium)	no, recruiting additional medical staff can be envisaged but more expensive to organize	no, recruiting additional medical staff can be envisaged but more expensive to organize
anonimity guaranteed	yes, due collaboration with CLB (in Belgium)	Other third party (such as the camp supervisors or scout leaders) could be envisaged but more challenging to organize	Other third party (such as the camp supervisors) could be envisaged but more challenging to organize
grouped in age categories	yes, with smaller age differences than in (2) and (3), more convenient for this type of study	usually broader age categories	usually broader age categories
flexible day time schedule to conduct study	No: class schedule to take into account	Uncertain: potential camp activities could interfere with the field study schedule	Uncertain: potential camp activities could interfere with the field study schedule
flexible period to conduct study	School holidays have to be taken into account, but study possible during large period in the year	No: fixed camp dates but only during (summer) school holidays	No: fixed camp dates, but only during school holidays
easy installation of monitoring station	depends on available space in the selected locations.	depends on available space in the selected locations. Specific electric requirements more difficult to meet when camp location is in woody area	depends on available space in the selected locations.

Table S3: Profile and micro-environment of the urban and rural school and possible surrounding emission sources.

Characteristics	Urban school	Rural school
type of street where school is located	street canyon, quite busy street and environment	along large/wide road, not so busy
type of area	densely populated, residential area	scarcely populated area with woods and farmland
type of building	low energy school	classic building
distance to highway or national road	450m (circular highway)	450m (national road)
distance to industry	250m from canal*(presence of construction companies and brewery, recycling companies along the canal)	8.5 km from industrial area

other aspects	motivated school, participation to other air quality campains. Recent action: reduced car passage during morning and evening rush hour	N/A

* measuring station located near canal

Table S4: **Summary of successful sampling, measurements and analyses of the different biomarkers**.

	**Urban**		**Rural**	
	**time point 1**	**time point 2**	**time point 1**	**time point 2**
**Measurements**				
Height and weight	20 (100%)	20 (100%)	20 (100%)	20 (100%)
Blood Pressure	18 (95%)	19 (100%)	23 (100%)	23 (100%)
FeNO	19 (100%)	19 (100%)	23 (100%)	21 (91%)
Lung function[c]	19 (100%)	19 (100%)	23 (100%)	23 (100%)
**Sampling**				
Urine				
Main collection	19 (100%)	19 (100%)	23 (100%)	23 (100%)
Additional collection[a]	3 (100%)	3 (100%)	3 (100%)	3 (100%)
Saliva				
saliva collection 1	19 (100(%)	19 (100(%)	22 (96%)	22 (96%)
saliva collection 2	19 (100(%)	19 (100(%)	22 (96%)	22 (96%)
saliva collection 3[b]	19 (100(%)	N/A	22 (96%)	N/A
**Biomarker investigation**				
**Genetic[g]**				
CC16 SNP G38A	19 (100%)	N/A	21 (95%)[c]	N/A
UGRP1 SNP G112A	19 (100%)	N/A	21 (95%)[c]	N/A
**Protein[h]**				
U-CC16 protein	18 (95%)	19 (100%)	21 (91%)	20 (87%)
U-β2M protein	18 (95%)	19 (100%)	22 (96%)	23 (100%)
U-RBP4 protein	18 (95%)	19 (100%)	22 (96%)	23 (100%)
**Epigenetic**				
**Methylation[di]**				
TET-1	3 (100%)	0	3 (100%)	0
**miRNA[ej]**				
miR-146a	1 (100%)	1 (100%)	1 (100%)	1 (100%)
miR-222	1 (100%)	1 (100%)	1 (100%)	1 (100%)
miR-191	1 (100%)	1 (100%)	1 (100%)	1 (100%)
cel-miR-39	1 (100%)	1 (100%)	1 (100%)	1 (100%)

The percentages (%) are expressed in function of total of received/analyzed items (not total children initially participating)

[a] Additional samples were colected from 3 children, randomly selected, during both phases of the study on the day of the field study conducted in the other area

[b] Not collected on the second time point

[c] from the 22 received saliva samples, one was not succesfully genotyped, due to technical issue during DNA extraction

[d] Analysis limited to randomly selected saliva samples from 3 children from urban school and 3 children from rural school, from whom a saliva sample was collected on time point 1 and time point 2

[e] Analysis limited to randomly selected saliva samples from one children from an urban school and one children from a rural school, from whom a saliva sample was collected on time point 1 and time point 2

[f] Measurement with nose clip challenging in children - repeated without nose clip

[g] Succesful analysis refers to the unbiased straightforward identification of the different genetic variants in high qualitative DNA samples using commercial genotyping assays [32]

[h] Succesful analysis refers to relative quantification of the different proteins using a validated MRM method, with values above the LOQ [19]

[i] Succesful analysis refers to quantification of the methylation level (in %) using validated 'gold standard' methods of bisulphite treatment followed by pyrosequencing [52]

[j] Succesful analysis refers to the relative detection of different miRNAs in salivary extracellular fraction using commercial kits [42], [68]

N/A: not applicable (not collected, not measured)

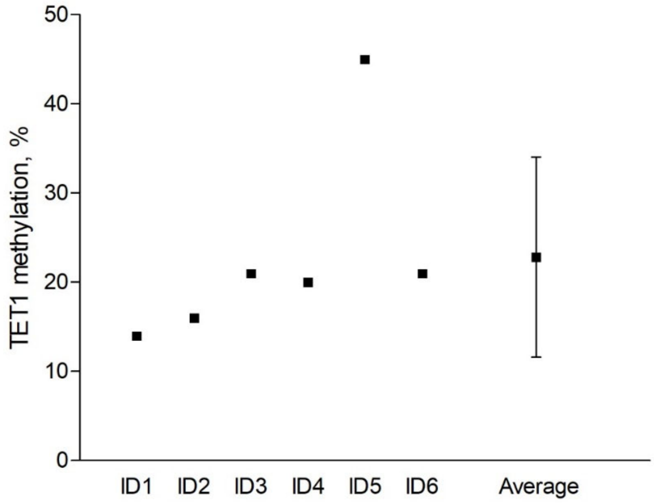

Figure S3: TET1 methylation (%) of 6 randomly chosen salivary DNA samples

Table S5: Cq-values (SD) representing the expression of miR-222 miR-146a, miR-191 (endogenous control) and cel-miR-39 (exogenous control) obtained by reverse transcription and quantitative real time PCR (RT-qPCR).

Cq (SD)	cel-miR-39	miR-146a	miR-191	miR-222
sample 1	13.92 (0.21)	22.82 (0.23)	19.07 (0.19)	19.74 (0.20)
sample 2	14.88 (0.57)	22.78 (0.61)	21.03 (0.45)	20.08 (0.51)
sample 3	13.98 (0.04)	23.32 (0.06)	19.74 (0.08)	20.76 (0.14)
sample 4	13.21 (0.17)	24.25 (0.24)	20.63 (0.13)	21.40 (0.16)
